# Hypercalcemia Caused by Metastatic Adamantinoma: Response to Radiotherapy

**DOI:** 10.1080/13577149977848

**Published:** 1999-03

**Authors:** Janice A. Lyons, G. Thomas Budd, Richard L. Crownover

**Affiliations:** 1 Department of Radiation Oncology The Cleveland Clinic Foundation Cleveland Ohio 44195 USA; 2 Department of Hematology Oncology The Cleveland Clinic Foundation Cleveland Ohio USA

## Abstract

*Purpose.* To describe successful palliation of a patient with metastatic adamantinoma presenting with lung metastases and hypercalcemia resulting from a parathormone-like substance released from the tumor.

*Methods and materials.* The records of a patient with a history of a tibial adamantinoma who presented with symptoms of hypercalcemia 20 years after the original surgery, as well as the literature concerning hypercalcemia and adamantinoma were reviewed and summarized.

*Results.* After thorough review of the literature we found no prior reports of radiation being used for palliation of hypercalcemia associated with metastatic adamantinoma.We report rapid improvement in symptoms and normalization of serum calcium levels following a course of radiation therapy. The patient remains asymptomatic 15 months following radiotherapy despite a gradual return of elevated serum calcium levels.

*Discussion.* Radiation therapy should be considered as a palliative option for patients who are not surgical candidates presenting with medically refractory hypercalcemia.


*Sarcoma* (1999) **3**, 33–35

ORIGINAL ARTICLE

### Hypercalcemia caused by metastatic adamantinoma: response to radiotherapy

JANICE A. LYONS,[1] G. THOMAS BUDD[2] & RICHARD L. CROWNOVER[1]

*Departments of [1]Radiation Oncology and [2]Hematology Oncology, The Cleveland Clinic Foundation, Cleveland, Ohio, USA*

**Abstract**

*Purpose.* To describe successful palliation of a patient with metastatic adamantinoma presenting with lung metastases and hypercalcemia resulting from a parathormone-like substance released from the tumor.

*Methods and materials.* The records of a patient with a history of a tibial adamantinoma who presented with symptoms of hypercalcemia 20 years after the original surgery, as well as the literature concerning hypercalcemia and adamantinoma were reviewed and summarized.

*Results.* After thorough review of the literature we found no prior reports of radiation being used for palliation of hypercalcemia associated with metastatic adamantinoma. We report rapid improvement in symptoms and normalization of serum calcium levels following a course of radiation therapy. The patient remains asymptomatic 15 months following radiotherapy despite a gradual return of elevated serum calcium levels.

*Discussion.* Radiation therapy should be considered as a palliative option for patients who are not surgical candidates presenting with medically refractory hypercalcemia.

**Key words**: *Adamantinoma, hypercalcemia, radiation, PTH-like hormone.*

### Introduction

Adamantinoma is a rare tumor accounting for approximately 0.1–0.3% of primary bone tumors.[1,2] It is a slow growing tumor and tends to be locally aggressive but rarely metastasizes.[3] The rate of metastasis is approximately 15–20% and usually occurs in the first two years following diagnosis.[4,5] The most common sites of metastases are bone, lung, and regional lymph nodes.[4] Although many patients with osteolytic bony metastases from other primary tumors present with hypercalcemia it is unusual to see hypercalcemia in patients with adamantinoma, especially in the absence of bony disease. Here we present a patient with an adamantinoma of the tibia who subsequently developed lung metastases and severe hypercalcemia.

### Case report

A 39-year-old white male presented in 1973, at the age of seventeen, with a left tibial mass. Biopsy revealed an adamantinoma. The patient subsequently underwent a below knee amputation. He did well until 1993 when he started experiencing increasing weakness and cough. Chest X-ray at that time revealed bilateral pulmonary nodules. Biopsy of a left lung nodule revealed metastatic adamantinoma. Magnetic resonance imaging (MRI#)0 of the abdomen was negative but images of the chest revealed a heterogeneous soft tissue mass in the left hemithorax enlarging the costophrenic sulcus, probably located in the pleural space and contiguous with both the diaphragm and visceral pleura of the paraspinal soft tissue. There was also a 2×3 cm left anterior chest wall mass and a right middle lobe nodule. The serum calcium was 12.8 mg/dl (normal range 8.5–10.5). He underwent right upper lobectomy and wedge resection of the right middle and lower lobes, confirming metastatic adamantinoma. This was followed by a wedge resection of the left upper lobe that was negative for neoplasm and resection of tissue adjacent to the aorta which revealed metastatic adamantinoma.

The patient was seen in Radiation Oncology following surgical resection; however, due to anticipated high dose and large field size requirements it was thought that chemotherapy was a better option. He received six cycles of chemotherapy with mesna, adriamycin, ifosfamide, and DTIC (MAID) with granulocyte colony-stimulating factor (G-CSF) and interleukin-6 (IL-6) support from October 1993 to February 1994. This was followed by six cycles of carboplatin from June 1994 to December 1994.

Correspondence to: Richard L. Crownover, Ph.D., M.D., Department of Radiation Oncology, Desk T-28, The Cleveland Clinic Foundation, Cleveland, OH 44195, USA. Tel.: (216) 444-1925; Fax: (216) 444-5331; email: crownover@radonc.ccf.org

1357-714X/99/010033-03 $9.00 © 1999 Taylor & Francis Ltd

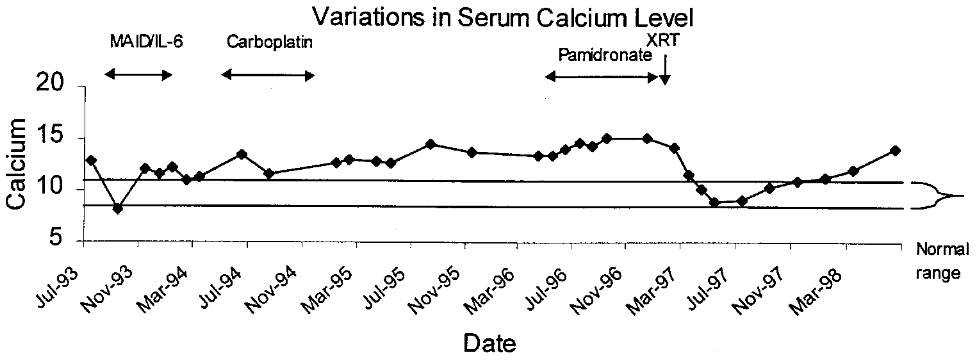

**Fig. 1.** *Variations in serum calcium levels during treatment.*

The patient was maintained on pamidronate for chronic tumor related hypercalcemia from April 1996 to December 1996 when computerized tomography (CT) scan of the chest revealed a large soft tissue mass in the left lower lobe of the lung involving the diaphragm and extending into the abdomen lateral to the spleen. There was rib involvement, displacement of the heart and esophagus to the right, encasement of the aorta and compression of the left atrium. At that time the patient was complaining of rib pain, dyspnea on exertion, fatigue and constant thirst. His calcium was 15.2 mg/dl. He was tested for parathormone related peptide and his serum level was elevated at 9.6 pmol/l (normal range 0.0–1.5).

The patient received radiation therapy to the left hemithorax from February 1997 until March 1997. This consisted of 45 Gy prescribed to the 98% isodose line via left posterior oblique/left anterior oblique field arrangement using 6 and 10 MV photons delivered by a linear accelerator equipped with a multi-leaf collimator. A partial transmission block was effected by varying the leaf positions during treatment to limit the dose to the left ventricle to 36 Gy. We used a lung correction factor of 0.33. The patient tolerated treatment well; however, he required a brief hospitalization for atrial fibrillation and pericarditis toward the end of the course. This resolved during his hospital stay. The patient's energy level increased during the course of treatment and his calcium level at completion was 11.5 mg/dl. The patient was able to work full time as a high-level software developer throughout the course of treatment. For 9 months following treatment the patient's calcium level remained around the normal range; however, recently this has started to increase with his last calcium being 14.0 mg/dl (June 1998; Fig. 1) He continues to work full time and is without complaints 15 months following completion of radiation.

### Discussion

Adamantinoma (ameloblastoma) is a rare entity that usually arises in the mandible but has been demonstrated in other bones. This tumor is considered to be low grade; however, it does tend to have a high local recurrence rate if marginal surgery is performed.[6] There have been several reports of patients with lung metastases that develop several years after the original diagnosis.[7–10] Since patients with metastatic disease can still have prolonged survival, aggressive therapy is often warranted.

Hypercalcemia is a common occurrence in cancer patients. It can result from bone destruction from metastatic disease or it may arise from tumoral secretion of parathormone-like substances. In some of these cases radiation therapy has been used sucessfully to control tumor-related hypercalcemia that is refractory to other therapy.[11–14]

There are reports in the literature of patients with metastatic ameloblastoma presenting with hypercalcemia; in these cases the hypercalcemia is often associated with high levels of a parathyroid-like hormone circulating in the blood.[15] Although this phenomenon is often associated with metastatic disease, there have been similar cases reported in which the primary tumor was thought to be producing a parathyroid-like substance. In one case, resection of the tumor led to normalization of the serum calcium level.[16] Our case demonstrates that radiation therapy can be used as another modality to normalize elevated calcium levels produced by metastatic adamantinoma and should be considered as a palliative treatment option in patients presenting with medically refractory hypercalcemia who are not surgical candidates.

### References

1 Mirra JM. Adamantinoma and osteofibrous dysplasia. In: *Bone tumors. clinical, radiologic, and pathologic correlations.* Philadelphia: Lea and Febiger, 1989: 1204–31.
2 Moon NF, Mori H. Adamantinoma of the appendicular skeleton—updated. *Clin Orthop* 1986; 204:215–37.
3 Gardner DG, Pecak AMJ. The treatment of ameloblastoma based on pathologic and anatomic principles. *Cancer* 1990; 46:2514–19.
4 Weiss SW, Dorfman MB. Adamantinoma of long bone. An analysis of nine new cases with emphasis on metastasizing lesions and fibrous dysplasia like changes. *Human Pathol* 1977; 8:141–53.
5 Altmannsberger M, Poppe H, Schauer A. An unusual case of adamantinoma of long bones. *J Cancer Res Clin Oncol* 1982; 104:315–20.

6 Jundt G, Remberger K, Roessner A, Schultz A, Bohndorf K. Adamantinoma of long bones. A histopathological and immunohistochemical study of 23 cases. *Pathol Res Practice* 1995; 191:112–20.

7 Sheppard BC, Temeck BK, Taubenberger JK, Pass HI. Pulmonary metastatic disease in ameloblastoma. *Chest* 1993; 104:1933–35.

8 Hazelbag HM, Taminiau AH, Fleuren GJ, Hogendoorn PC. Adamantinoma of the long bones. A clinicopathological study of thirty-two patients with emphasis on histological subtype, precursor lesion, and biological behavior. *J Bone Joint Surg—Am Vol* 1994; 76:1482–99.

9 Inoue N, Shimojyo M, Iwai H, *et al*. Malignant ameloblastoma with pulmonary metastasis and hypercalcemia. Report of an autopsy case and review of the literature. *Am J Clin Pathol* 1988; 90:474–81.

10 Harada K, Suda S, Kayano T, Nagura H, Enomoto S. Ameloblastoma with metastasis to the lung and associated hypercalcemia. *J Oral Maxillofac Surg* 1989; 47:1083–87.

11 Neskovic-Konstantinovic Z, Susnjar S, Vasovic S, *et al*. Tumour-induced hypercalcemia, resistant to systemic anti-hypercalcemic and chem-endocrine treatments, but responding to radiotherapy in a breast cancer patient. *Acta Oncol* 1996; 35:501–503.

12 Suzuki K, Tanaka H, Shibusa T, *et al*. Parathyroid-hormone-related-protein-producing thymic carcinoma presenting as a giant extrathoracic mass. *Respiration* 1998; 65:83–85.

13 Al-Rashid RA, Cress C. Hypercalcemia associated with neuroblastoma. *Am J Dis Child* 1979; 133:838–841.

14 Bakri YN, Akhtar M. Gonadal dysgerminoma-seminoma associated with severe hypercalcemia. *Acta Obstet Gynecol Scand* 1993; 72:57–59.

15 Madiedo G, Choi H, Kleinman JG. Ameloblastoma of the maxilla with distant metastases and hypercalcemia. *Am J Clin Pathol* 1981; 75:585–91.

16 McGuirt WF, Scruggs MS, Koufman JA. Hypercalcemia secondary to a pseudoparathormone-secreting ameloblastoma. *Arch Otolaryngol* 1981; 107:487–90.
